# The Scent of the Waggle Dance

**DOI:** 10.1371/journal.pbio.0050228

**Published:** 2007-08-21

**Authors:** Corinna Thom, David C Gilley, Judith Hooper, Harald E Esch

**Affiliations:** 1 Arizona Research Laboratories, Division of Neurobiology, University of Arizona, Tucson, Arizona, United States of America; 2 Carl-Hayden Bee Laboratory, United States Department of Agriculture, Tucson, Arizona, United States of America; 3 Department of Biology, University of Notre Dame, Notre Dame, Indiana, United States of America; University of London, United Kingdom

## Abstract

The waggle dance of honey bee (Apis mellifera L*.*) foragers communicates to nest mates the location of a profitable food source. We used solid-phase microextraction and gas chromatography coupled with mass spectrometry to show that waggle-dancing bees produce and release two alkanes, tricosane and pentacosane, and two alkenes, Z-(9)-tricosene and Z-(9)-pentacosene, onto their abdomens and into the air. Nondancing foragers returning from the same food source produce these substances in only minute quantities. Injection of the scent significantly affects worker behavior by increasing the number of bees that exit the hive. The results of this study suggest that these compounds are semiochemicals involved in worker recruitment. By showing that honey bee waggle dancers produce and release behaviorally active chemicals, this study reveals a new dimension in the organization of honey bee foraging.

## Author Summary

A honey bee colony consists of many thousands of individuals, all of which help to perform the work that allows their colony to thrive. To coordinate their efforts, honey bees have evolved a complex communication system, no part of which is more sophisticated than the waggle dance. The waggle dance is unique, because it exhibits several properties of true language, through which a forager communicates the location and profitability of a food source to other bees in the darkness of the hive. The information coded in the dance has been extensively researched, but we still do not understand how information is actually transferred from the dancing bee to the receivers of the message. Because information is often transferred by scent in honey bee colonies, we investigated whether waggle dancers produce a scent that distinguishes them from foragers that do not dance. We found that dancers produce four hydrocarbons that distinguish them from nondancing foragers, and that, when blown into the hive, increase foraging activity. These results show that waggle-dancing bees produce a unique scent that affects the behavior of their fellow foragers. We discuss likely meanings of this olfactory message and its potential role in waggle-dance communication.

## Abstract 3

Waggle-dancing honey bees, in addition to attracting nest mates to a food source, also release a scent that recruits more workers to the advertised site.

## Introduction

More than fifty years ago, Karl von Frisch demonstrated through a series of elegant experiments that the waggle dance of honey bees uses symbolic communication to convey information about a subject that is both spatially and temporally removed from the receiver of the signal [1]. The waggle dance is therefore a unique animal signal that exhibits several of the important properties of true language, which are generally attributed only to “advanced” organisms such as marine mammals, nonhuman primates, and humans. Honey bees are themselves quite advanced, however; a honey bee society consists of a large group of individuals of overlapping generations, living permanently together with cooperative care of young and a division of labor. To coordinate the complex interactions among the members of such a society, a sophisticated system of communication is necessary.

The role of the waggle dance in this sophisticated system of communication is primarily to direct the colony's foraging effort toward nectar- and pollen-producing flowers. Successful foragers perform the dance within the nest to recruit other bees to a profitable food source. By closely following a dancer, potential recruits acquire information about the location and richness of the advertised food source [1,2]. However, despite our considerable knowledge of the information contained in the dance, we still do not understand how dancers attract and convey information to recruits in the darkness of the hive [2]. Airborne sounds [3,4], substrate vibrations [5,6], and tactile cues [7] seem to play some role in attracting recruits to waggle dancers or conveying dance information, but each of these modalities appears to be neither necessary nor sufficient for recruitment [2].

Another modality that may be involved in waggle dance communication is olfaction. Evidence to date that olfaction plays a role in honey bee waggle-dance communication is limited to odors acquired from the environment at or en route to the floral food source [2,8,9]. (These odors are thought to serve as cues that allow foraging recruits to pinpoint the food source advertised by the waggle dancer.) However, the production of olfactory signals by the waggle dancers themselves could attract recruits and convey dance information. Pheromones and other semiochemicals are used frequently by honey bees and other social insects to coordinate the activities of colony members [10,11] and hence may be expected to help organize the vital task of foraging. Furthermore, evidence suggests that closely related Hymenopterans, such as bumblebees, use pheromones within the nest to organize foraging activity [12]. Whereas bumblebees do not communicate via waggle dances, olfactory-based foraging communication may be an ancestral trait and thus present in honey bees. A preliminary study suggested indeed that the scent of active honey bee foragers could encourage other bees to forage [13].

The goal of this study was to investigate whether waggle dancers produce and release into the air chemical compounds that distinguish them from other foragers. We addressed this first goal by using solid phase microextraction (SPME) and gas chromatography coupled with mass spectrometry (GC/MS). If these distinguishing compounds are semiochemicals or pheromones, then adding these compounds into the hive will affect the behavior of foraging bees. To test whether the compounds are behaviorally active, we measured foraging activity before and after we injected the volatilized compounds into the hive. Our results show that honey bee waggle dancers produce four characteristic volatile compounds that increase foraging activity.

## Results

**Figure 1:**
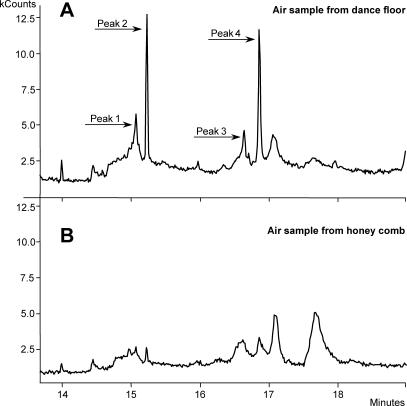
Comparison of Air Samples above Waggle Dancers and Nondancing Bees A chromatogram of an air sample taken from the dance-floor area on the bottom hive frame, where waggle dancers were present (A) and of a sample from the top frame, where honey was stored and no waggle dancers (but other bees) were present (B). Relative amount of fragments is measured in thousands of counts. Peaks 1, 2, 3, and 4 are Z-(9)-tricosene, tricosane, Z-(9)-pentacosene and pentacosane, respectively, and these peaks distinguish the air over dancing bees from the air over nondancing bees.

We discovered four conspicuous compounds in the airspace surrounding dancing bees that were not present in the airspace surrounding nondancing bees (Figure1). We obtained similar results for the airspace surrounding waggle dancers on an artificial swarm as compared with the air over an area of the swarm with no waggle dancers. From extracts made of three intact waggle dancers immersed for 1 min into 250 μl of hexane, we identified the four substances that generated peaks 1, 2, 3, and 4 as marked in Figure1 to be Z-(9)-tricosene, tricosane, Z-(9)-pentacosene, and pentacosane, respectively.

**Figure 2:**
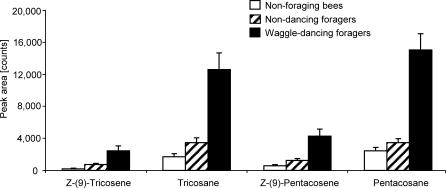
Relative Abundance of Alkanes and Alkenes Relative abundance of Z-(9)-tricosene, tricosane, Z-(9)-pentacosene, and pentacosane sampled from three types of bees. The height of each bar indicates the mean peak area for each compound from GC/MS chromatograms. The relative abundance of compounds is significantly higher for waggle-dancing foragers (black bars) than for either nondancing foragers (hatched bars) or nonforaging bees (white bars, for statistical tests, see text). The relative abundance of compounds for nondancing foragers does not differ from that for nonforaging bees, with the exception of Z-(9)-tricosene. Error bars represent the standard error of the average of six daily mean values, each daily mean comprising a sample of two–three bees of each type. Peak areas represent relative abundance only and thus do not enable comparison among different compounds.

**Figure 3:**
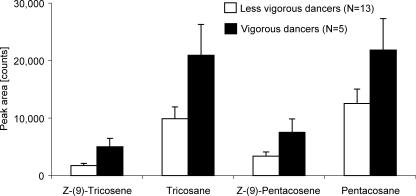
Comparison of Vigorous and Less-Vigorous Dancers The relative abundance for Z-(9)-tricosene, tricosane, Z-(9)-pentacosene, and pentacosane tended to be higher for vigorous (*n* = 5, black bars) than less-vigorous (*n* = 13, white bars) dancers. Vigor of dancing was assessed subjectively by one experimenter and included appearance of abdomen movement and the rate of waggle runs. More vigorous dancers appeared to move their abdomen farther to the side and perform more waggle runs than less-vigorous dancers. Error bars represent the standard error. Statistical tests are given in the text.

All four compounds were present in significantly higher amounts on the abdomens of waggle dancers than in either (a) nondancing foragers that returned from the same unscented nectar source, or (b) nonforaging bees [Figure 2, one-way analysis of variance (ANOVA) for each compound, degrees of freedom (df) = 2, 49, *p* < 0.001 for all compounds; Tukey HSD for unequal *n*, *p* < 0.0005 for all compounds, experiment-wise α = 0.05]. Intriguingly, although only marginally significant, waggle dancers that danced more vigorously (i.e., appeared to perform waggle runs at higher rates and with more exaggerated movement of the abdomen as assessed subjectively by one person, *n* = 5 bees) tended to emit more of all four compounds than less vigorous dancers did (*n* = 13) (Figure 3, Mann-Whitney *U* test, *p* = 0.05, 0.07, 0.13, and 0.08 for peaks 1, 2, 3, and 4, respectively). Nondancing foragers and nonforaging bees did not emit different amounts of these compounds (Tukey HSD for unequal *n*, *p* = 0.076, 0.074, and 0.400, for peaks 2, 3, and 4, respectively) with the exception of Z-(9)-tricosene (*p* < 0.001).

To test whether the waggle-dance–specific substances affect behavior, we injected onto the dance floor a gaseous mixture of the three commercially available compounds dissolved in hexane [hereafter called TTP (Z-(9)-tricosene, tricosane, pentacosane) solution]. TTP trials lasted 32 min, during which we recorded the number of bees exiting the hive each minute, and during which we made two injections: Injection 1, which consisted of 50 μl of pure hexane, was made during minute 1, and Injection 2, which consisted of 50 μl of TTP solution, was made during minute 16. To control for solvent and treatment, we performed “Hexane trials”, in which Injection 2, like Injection 1, consisted of 50 μl of pure hexane. Experiments were done with two colonies, C1 and C2, using one colony at a time. We performed a total of 20 trials (10 TTP trials and 10 Hexane trials) with C1, and a total of 28 trials (15 TTP trials and 13 Hexane trials) with C2. Only one trial was performed per day, and all trials were performed at the same time of day. Because the compounds originate from waggle dancers under natural conditions, we ascertained that at least one dancer was present during each trial.

**Figure 4:**
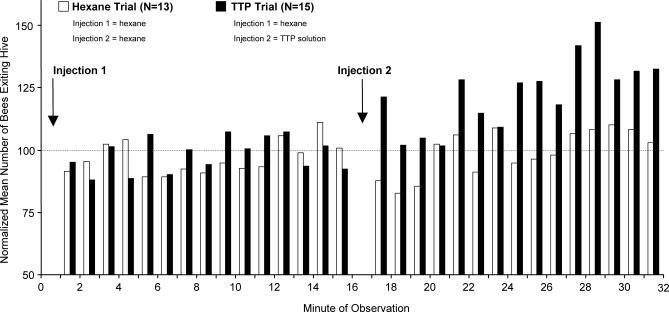
Injection of Scent and Behavioral Response Comparison of foraging activity in Colony 2 following injection of the TTP solution versus pure hexane. The normalized mean number of bees exiting the hive following Injection 2 (during minutes 25–32) was greater during TTP trials (black bars) than during Hexane trials (white bars). Results were similar for Colony 1. Statistical tests are given in the text.

Injection of TTP increased the number of bees exiting the hive (Figure 4). The normalized mean number of bees exiting the hive during minutes 25–32 differed significantly between TTP trials (i.e., Injection 2 = TTP solution) and Hexane trials (i.e., Injection 2 = hexane) (two-sample *T*-test on normally distributed sample groups with equal variances; Colony 1: *T* = −3.29, *p* = 0.004, df = 18; Colony 2: *T* = −2.25, *p* = 0.033, df = 26). This difference between TTP trials and Hexane trials was not observed following Injection 1, which consisted of hexane for both types of trial (Colony 1: *T* = −0.61, *p* = 0.551, df = 18; Colony 2: *T* = −0.47, *p* = 0.643, df = 26).

We also observed within TTP trials an increase in the number of bees exiting the hive following Injection 2, as compared with the number exiting following Injection 1, but this was significant for only one colony (two-sample *T*-test; Colony 1: *T* = −1.63, *p* = 0.121, df = 18; Colony 2: *T* = −2.66, *p* = 0.013, df = 28). This increase could possibly be the result of circadian foraging patterns, but this would not explain the lack of such an effect during Hexane trials (two-sample *T*-test; Colony 1: *T* = 0.83, *p* = 0.415, df = 18; Colony 2: *T* = −0.68, *p* = 0.502, df = 24), which were conducted at the same time of day as TTP trials.

## Discussion

We have identified in this study four compounds that are unique to waggle-dancing bees and that are behaviorally active. This waggle-dance scent originates from the waggle dancers themselves; it is not acquired from the environment while foraging, nor is it a byproduct of a bee's age or task, because it is emitted in only minute quantities by nondancing foragers returning from the same food source.

The prominent compounds of the waggle-dance scent are the cuticular hydrocarbons Z-(9)-tricosene, tricosane, Z-(9)-pentacosene, and pentacosane. Hydrocarbons such as these are produced subcutaneously and are not stored within a gland [14]. The waggle-dance scent is therefore best classified as a semiochemical or nonglandular pheromone. Waggle dancers could raise the levels of the compounds through increased synthesis and/or increased release onto the epicuticle. The compounds could then be released passively into the air, perhaps assisted by the relatively high body temperature of the waggle dancers.

The chemical nature and source of the compounds of the waggle-dance scent differ from those of the bumblebee foraging recruitment pheromone [15]. The main compounds of the bumblebee recruitment pheromone were identified as eucalyptiol, ocimene, and farnesol, which are terpene derivatives. These compounds are produced in the bees' tergal glands. Their different chemistry and source suggest different evolutionary origins for the bumblebee foraging recruitment pheromone and the waggle-dance scent of honey bees. Hence, it is unlikely that the waggle-dance scent of honey bees has evolved from the bumblebee foraging recruitment pheromone.

The compounds of the waggle-dance scent have been identified in earlier studies on honey bees. Z-(9)-tricosene, tricosane, Z-(9)-pentacosene, and pentacosane have been previously identified in hexane washes of the cuticles of foraging-age worker bees [16] and have been shown to be perceptible to honey bee workers [17]. More recent work has shown that, among many others, the four compounds are present in the air surrounding foragers at a feeder station [18]. However, with the exception of demonstration of a possible kin-recognition function of Z-(9)-tricosene [19], past studies did not link specific compounds with specific behaviors of honey bees. In insects other than honey bees, however, the individual compounds of the waggle-dance scent have been linked to specific behaviors. For example, tricosane, pentacosane, and Z-(9)-pentacosene are compounds of a pheromone that foragers of the social wasp Vespula vulgaris use to lay and follow terrestrial trails [20]. Compounds of the waggle-dance scent are also well-known sex attractants in other insects, such as flies [14].

The waggle-dance scent may have a similar marking or attracting function, which the context and results of our experiments link to recruitment behavior. Honey bees produce the scent when they perform waggle dances, both in the hive and on swarms, and in both contexts, workers are recruited to the advertised site. In the more common context of foraging, a general measure of recruitment is the number of foragers that leave the hive. Our results show that injection of the waggle-dance scent onto the dance floor increased the number of bees that left the hive. These bees can be assumed to be foragers, because only foragers leave the hive without noticeably hesitating, performing orientation flights, or gathering at the hive entrance.

Our experiments show that the waggle-dance scent increases the number of foragers leaving the hive, but the exact mechanism underlying the effect is still unclear. Given the function of the waggle-dance scent in other hymenoptera, we propose that the waggle-dance scent, which in our experiments was fanned onto the dance floor, attracts potential recruits to the dance floor, thereby increasing the probability of encounters between potential recruits and dancers, and finally the number of recruits. Under natural conditions, the scent would originate from the dancers themselves, thus the odor plume would mark not only the dance floor, but the individual dancers. This should enable recruits to locate the dancers themselves, which could enhance recruitment efficiency. Recruits could even seek out more vigorous dancers, who typically advertise especially profitable food sources [21], and who seem to emit higher concentrations of the scent. Hence, the waggle-dance scent may mark and attract recruits to successful foragers, and thus help to rapidly focus the colony's foraging effort on the most profitable food sources. Besides marking successful dancers, it is feasible that the waggle-dance scent facilitates the transfer of information from the dancer to recruits. The spatio-temporal pattern of a dancer's odor plume could, for example, indicate the length of a waggle run, and thus provide information to a recruit, even if she lost contact with the dancer herself. This hypothesis is supported by the observation that a mechanical model of a waggle dancer recruits bees to a food source only after the model has touched a waggle dancer (H. E. Esch, personal observation).

Whereas the waggle-dance signal is likely a signal intended for new recruits, two other groups of bees, namely foragers already devoted to a food source and in-hive receiver bees, could glean cues from the waggle-dance scent. Foragers that are already devoted to a food source do not readily follow new dances if their source becomes unavailable, but rather wait for it to replenish [8]. Because the waggle-dance scent does not seem to identify specific food sources, it can provide only limited information to these foragers. However, high concentrations of the scent could alert them to generally good foraging conditions. This could be useful at the beginning of daily foraging or if foraging can be resumed after a spell of bad weather. This mechanism may have been responsible for the effect observed in an earlier preliminary study, in which the number of foragers visiting an empty feeder increased following exposure to the air from a foraging colony [13]. In our experiments, however, the increase in foragers was not likely caused by already-devoted foragers for three reasons. First, experiments were done well after the time that colonies started daily foraging. Second, external conditions such as the weather were remarkably stable, which made strong fluctuations in the numbers of already-devoted foragers unlikely. Third, we did not record a conspicuous drop in the number of bees that left the hive after the initial increase (Figure 4); if the increase would have been due mostly to already-devoted foragers, we would have expected a quick drop to original levels once these foragers found that there was no change in food-source profitability. However, it is possible that such a drop could be hidden by the more substantial numbers of newly recruited foragers. Receiver bees are the second group of bees that could glean cues from the waggle-dance scent. Receiver bees unload nectar from newly returned foragers, which relieves foragers of the time-consuming search for empty storage cells. A high concentration of the waggle-dance scent would indicate a high demand for receiver bees, and could help attract receiver bees to the dance floor. The tremble dance, which is performed by successful foragers that perceive a shortage in receiver bees [22], may help to additionally spread the scent to potential receiver bees.

## Materials and Methods

### Bees.

Bees were kept indoors in four-frame observation hives at the Carl Hayden Bee Research Center in Tucson, Arizona, United States. To test whether waggle dancers produce specific chemical compounds, we performed experiments with two colonies, using first one, then the other colony. Foragers were trained to an artificial feeder [1] ∼100 m from the hive that offered nonscented sugar water. Foragers visiting the feeder were marked on the thorax with powdered paint and thus could be recognized in the hive.

### Sampling of chemical profiles.

We used a SPME fiber (65 μm polydimethylsiloxane/divinylbenzene; Supelco; http://www.sigmaaldrich.com/Brands/Supelco_Home.html) to sample chemicals. After sampling, the SPME fiber was injected into a GC, CP-3800 (Varian; http://www.varian.com) coupled to a MS (Saturn 2200 Ion Trap,Varian).

We compared the chemical profile of the air over dancing bees to that over nondancing bees, and we also compared the chemical profile of waggle dancers' abdomens to those of both nondancing foragers and nonforaging bees. For air samples, we exposed the fiber for 5 consecutive min approximately 2 cm above the surface of the comb. For abdomen profiles, we briefly touched the fiber to the tip of the abdomen of an individual bee [23–26]. Waggle dancers were sampled during a waggle run, shortly after they began dancing. Sampled bees were observed from the moment they entered the hive until the sample was taken. It is possible that we classified as nondancing foragers bees that danced before observation began (e.g., in the entrance tunnel to the hive) or that danced after SPME sampling, but this would bias our results in only a conservative direction.

### Compound identification.

After SPME sampling, the fiber was desorbed for 3 min in the GC/MS, and compounds separated on a Varian VF-5MS 30 m × 0.25 mm inner diameter (ID) column with an injector temperature of 250 °C, and a column temperature of 40 °C for 5 min, which was then ramped at 50 °C/min to 150 °C, followed by a ramp at 15 °C/min to 260 °C with a 4.5-min hold; flow rate was 1 ml/min. The MS was operated in electron ionization mode at 150 eV. Tentative identification of the peaks was made by comparing MS fragment patterns with spectra from the National Institute of Standards and Technology (NIST) 98 and Wiley library databases. Chemical ionization with acetonitrile was used to determine molecular weights and to assign double-bond position through derivatization of the double bond and formation of characteristic addition compounds [27,28]. The identities of peaks 1, 2, and 4 were further confirmed by the production of identical retention times and fragment patterns in both electron and chemical ionization modes when compared with chemical standards.

### Behavioral tests.

TTP trials consisted of 32 min during which we made two injections: Injection 1, 50 μl of pure hexane, during minute 1; Injection 2, 50 μl of TTP solution, during minute 16. Hexane trials were similar to TTP trials except that Injection 2, like Injection 1, consisted of 50 μl of pure hexane.

The TTP solution contained Z-(9)-tricosene, tricosane, and pentacosane, each diluted 1:100 in hexane and mixed at a ratio of 1:2:3, respectively (this ratio produced chromatograms with peak heights that approximately matched those from samples of waggle dancers), and then further diluted 1:10 in hexane. To volatilize the liquid TTP solution, we injected it into a heated glass tube with 0.5-cm ID. Immediately after injection, we inserted into the tube a fan to blow the vaporized solution through the tube and into a funnel (10-cm diameter) positioned 1 cm above the comb surface of a colony's dance floor. The identical method was used to volatilize and deliver hexane only during Hexane trials (Injection 1: hexane, Injection 2: hexane). To avoid contamination between TTP and Hexane trials, we used separate equipment for TTP solution and hexane. The temperature of the gaseous mixture arriving on the dance floor was maintained between 35 °C and 40 °C. At least one waggle dancer was present on the dance floor during each trial.

We conducted each trial on a different day between 14 July and 8 October 2004. To avoid seasonal effects, we randomized the type of trial (TTP or Hexane) performed each day. To reduce the effect of time of day, trials for a colony started during the same hour every day. Trials for the first colony started between 1200 and 1300, and for the second colony between 1030 and 1130. To further account for day and time effects, the data for each trial were normalized by dividing each 1-min count by the average number of bees exiting the hive per minute during the 10 min immediately preceding the trial.

### Statistics.

To compare the presence of the four compounds on the abdomens of waggle dancers with either nondancing foragers that returned from the same unscented food source or nonforaging bees, we used a one-way ANOVA for each compound using Box-Cox transformed data, and a Tukey HSD for unequal *n*, with data for both colonies pooled.

To compare the effect of injection of TTP solution with injection of hexane, we used two-sample *T*-tests on normally distributed sample groups with equal variances. To account for day and time effects, the data for each trial were normalized by dividing each 1-min count by the average number of bees exiting the hive per minute during the 10 min immediately preceding the trial.

## Abbreviations

df: degrees of freedom
GC: gas chromatography
MS: mass spectrometry
SPME: solid phase microextraction
TTP: Z-(9)-tricosene, tricosane, pentacosane

## References

[pbio-0050228-b001] von Frisch K (1965). Tanzsprache und Orientierung der Bienen.

[pbio-0050228-b002] Dyer FC (2002). The biology of the dance language. Annu Rev Entomol.

[pbio-0050228-b003] Kirchner WH, Summer K (1992). The dance language of the honey bee mutant diminutive wings. Behav Ecol Sociobiol.

[pbio-0050228-b004] Michelsen A, Andersen BB, Kirchner WH, Lindauer M (1989). Honeybees can be recruited by means of a mechanical model of a dancing bee. Naturwissenschaften.

[pbio-0050228-b005] Tautz J (1996). Honey bee waggle dance: Recruitment success depends on the dance floor. J Exp Biol.

[pbio-0050228-b006] Tautz J, Rohrseitz K (1998). What attracts honey bees to a waggle dancer?. J Comp Physiol A.

[pbio-0050228-b007] Rohrseitz K, Tautz J (1999). Honey bee dance communication: Waggle run direction coded in antennal contacts?. J Comp Physiol A.

[pbio-0050228-b008] Seeley TD (1995). The wisdom of the hive.

[pbio-0050228-b009] Farina WM, Grüter C, Díaz PC (2005). Social learning of floral odours inside the honeybee hive. Proc R Soc Lond B.

[pbio-0050228-b010] Free JB (1987). Pheromones of social bees.

[pbio-0050228-b011] Hölldobler B, Wilson EO (1990). The ants.

[pbio-0050228-b012] Dornhaus A, Brockmann A, Chittka L (2003). Bumble bees alert to food with pheromone from tergal gland. J Comp Physiol A.

[pbio-0050228-b013] Thom C, Dornhaus A (2007). Preliminary report on the use of volatile compounds by foraging honey bees in the hive (Hymenoptera: Apidae: Apis mellifera). Entomol Gen.

[pbio-0050228-b014] Howard RW, Blomquist GJ (1982). Chemical ecology and biochemistry of insect hydrocarbons. Annu Rev Entomol.

[pbio-0050228-b015] Mena Granero A, Guerra Sanz JM, Egea Gonzalez FJ, Martinez Vidal JL, Dornhaus A (2005). Chemical compounds of the foraging recruitment pheromone in bumblebees. Naturwissenschaften.

[pbio-0050228-b016] Blomquist GJ, Chu AJ, Remaley S (1980). Biosynthesis of wax in the honeybee, Apis mellifera L.. Insect Biochem.

[pbio-0050228-b017] Getz WM, Smith KB (1987). Olfactory sensitivity and discrimination in the honey bee Apis mellifera. J Comp Physiol A.

[pbio-0050228-b018] Schmitt T, Herzner G, Weckerle B, Schreier P, Strohm E (2007). Volatiles of foraging honeybees Apis mellifera L. (Hymenoptera: Apidae) and their potential role as semiochemicals. Apidology.

[pbio-0050228-b019] Breed MD (1998). Recognition pheromones of the honey bee, the chemistry of nest mate recognition. Bioscience.

[pbio-0050228-b020] Steinmetz I, Schmolz E, Ruther J (2003). Cuticular lipids as trail pheromone in a social wasp. Proc R Soc Lond B.

[pbio-0050228-b021] Seeley TD, Mikheyev AS, Pagano GJ (2000). Dancing bees tune both duration and rate of waggle-run production in relation to nectar-source profitability. J Comp Physiol A.

[pbio-0050228-b022] Seeley TD (1992). The tremble dance of the honey bee: Message and meanings. Behav Ecol Sociobiol.

[pbio-0050228-b023] Liebig J, Peeters C, Oldham NJ, Markstaedter C, Hölldobler B (2000). Are variations in cuticular hydrocarbons of queens and workers a reliable signal of fertility in the ant Harpegnathos saltator?. Proc Natl Acad Sci U S A.

[pbio-0050228-b024] Augusto F, Valente ALP (2002). Applications of solid-phase microextraction to chemical analysis of live biological samples. Trends Anal Chem.

[pbio-0050228-b025] Tentschert J, Bestmann HJ, Heinze J (2002). Cuticular compounds of workers and queens in two Leptothorax ant species – a comparison of results obtained by solvent extraction, solid sampling, and SPME. Chemoecology.

[pbio-0050228-b026] Dietemann V, Peeters C, Liebig J, Thivet V, Hölldobler B (2003). Cuticular hydrocarbons mediate discrimination of reproductives and nonreproductives in the ant Myrmecia gulosa. Proc Natl Acad Sci U S A.

[pbio-0050228-b027] Monetti G, Pieraccini G, Favretto D, Traldi P (1999). Reactions of ionic species from acetonitrile with long-chain saturated and unsaturated alcohols. J Mass Spectrom.

[pbio-0050228-b028] Oldham NJ (1999). Ion/molecule reactions provide new evidence for the structure and origin of [C3H4N]+ from acetonitrile chemical ionization plasma. Rapid Commun Mass Spectrom.

